# Impact of orthodontic treatment with aligners and fixed appliances on OHRQoL: a randomized clinical trial

**DOI:** 10.1590/1807-3107bor-2025.vol39.012

**Published:** 2025-02-07

**Authors:** Thais Teixeira BORSATO, Jéssica Madeira BITTENCOURT, Saul Martins PAIVA, Ana Cláudia de Castro Ferreira CONTI, Thais Maria Freire FERNANDES, Renata Rodrigues de ALMEIDA-PEDRIN, Marcio Rodrigues de ALMEIDA, Paula Vanessa Pedron OLTRAMARI

**Affiliations:** (a) Universidade Uniderp, Department of Orthodontics, Campo Grande, MS, Brazil.; (b) Universidade Federal de Minas Gerais – UFMG, School of Dentistry, Department of Pediatric Dentistry and Orthodontics, Belo Horizonte, MG, Brazil.

**Keywords:** Orthodontics, Quality of Life

## Abstract

The aim of this study was to assess the impact of orthodontic treatment with orthodontic aligners (OAs) and fixed appliances (FAs) on oral health-related quality of life (OHRQoL). This parallel randomized clinical trial included 40 male and female patients aged 13 to 35 years diagnosed with Angle’s Class I malocclusion. Participants were assigned to two groups: OA (n = 20) and FA (n = 20). OHRQoL was assessed using the Brazilian OHIP-14, which was administered before treatment (T0), at 1 month (T1), 6 months (T2), and 12 months (T3) after treatment initiation. Data were analyzed using the independent t test, the chi-square test, the Mann-Whitney test, and Friedman test (p < 0.05). FAs had a significantly (p < 0.05) higher impact on OHRQoL at T1 in terms of functional limitation, physical pain, psychological discomfort, physical disability, psychological disability, and overall score. Within-group comparison showed higher scores for the FA group in comparison to the OA group. Functional limitation scores were higher at T1 than at T0 (p = 0.034), while physical pain scores were higher at T1 compared to T0 (p = 0.034) and T2 (p = 0.010). Psychological discomfort scores were higher at T1 than at T2 (p = 0.015). Physical disability scores were higher at T1 compared to T0 (p = 0.008). Overall scores were higher at T1 than at T2 (p = 0.003). No significant changes were observed in the OA within-group comparison. Patients treated with OAs had less impact on OHRQoL compared to those treated with FAs in the first month. There was no difference between the groups at the 6-month follow-up.

## Introduction

Inclusion of a patient-centered approach has been explored in the context of a variety of oral conditions to complement the clinical examination performed by the dentist.^1^One of these approaches, the oral health-related quality of life (OHRQoL) is an extensively researched construct,^1^ which addresses individuals’ perception of the impact of their oral condition on everyday aspects of life.^1^ Malocclusion is one of the most significant oral conditions that can adversely affect individuals’ OHRQoL.^2,3^ Accordingly, individuals seek orthodontic treatment with the aim of improving their oral function, psychosocial well-being and, consequently, their OHRQoL. Moreover, improvement in appearance is one of the major motivations for orthodontic treatment among adult patients.^4^

The effects of fixed appliances (FAs) on orthodontic treatment have been widely investigated, and their efficacy has been proven.^5,6^ However, patients’ demands for more aesthetic, comfortable, and easy-to-clean appliances has increased in recent years.^7^ Thus, discomfort and concern about the appliance may affect patients’ attitude and compliance with treatment.^8^ Orthodontic aligners (OAs) represent an alternative to FAs for patients in terms of aesthetics, ease of cleaning, and greater comfort.^9,10^

A longitudinal study monitoring the first week of treatment showed that individuals treated with OAs experienced less pain and reported a reduced negative impact on their lives compared to those treated with FAs.^11^ A cross-sectional observational study ascertained that patients treated with OAs found it easier to chew than those treated with FAs.^12^ A prospective clinical study conducted over a period of 10 months evaluated adult patients wearing FAs, fixed lingual retainer, and OAs.^13^ The authors observed higher OHRQoL in the aligner group, followed by the lingual retainer and conventional FA groups. On the other hand, a randomized clinical study reported that the intensity of orthodontic pain, usually mild, was similar in the groups treated with FAs and OAs.^14^

The literature still lacks randomized clinical studies investigating OHRQoL in connection with the use of these two appliances. The aim of this study was to prospectively compare the impact of orthodontic treatment with OAs and FAs on OHRQoL. It was hypothesized that FA treatment would negatively impact participants’ OHRQoL compared to OA treatment.

## Methods

### Study design and data collection

This was a parallel randomized clinical trial in which participants were recruited prospectively. A total of 2,662 participants underwent eligibility verification after widespread outreach through social media, schools, and universities in the city of Londrina, Brazil, in June 2019. No changes were made in the methodology after commencement of the trial. The present study followed the Consort guidelines, which highlight the importance of sample calculation, inclusion of a control group, and randomization. These practices help minimize selection bias and are therefore fundamental components of high-impact RCTs.^15^

### Participants, eligibility criteria, and ethical aspects

The study population consisted of male and female individuals aged 13 to 35 years diagnosed with Angle’s Class I malocclusion and moderate mandibular anterior crowding who had not undergone any treatment with tooth extraction. Participants with missing permanent teeth, anterior or posterior open bite, anterior or posterior crossbite, and a history of prior orthodontic treatment were excluded.

The study protocol was approved by the Research Ethics Committee of Unopar (CAAE: 12088219.0.0000.0108) and was registered with the Brazilian Clinical Trials Registry (ReBEC: RBR-9zytwf). Written informed consent was obtained from all participants or their legal guardians prior to the study. Orthodontists supervised by an Orthodontics professor with over 15 years of experience performed the treatment on the participants.

### Study interventions

All patients included in the study completed the initial orthodontic work-up and answered the OHRQoL questionnaire.

Patients were randomly assigned to two groups:

- OA, orthodontic aligners (Smart Track, InvisalignTM, Align Technology, Santa Clara, USA): virtual planning conducted with ClinCheckTM Pro program, version 5.6 (Align Technology). The sequence of treatment procedures with aligners followed the outlined virtual plan. The pairs of upper and lower OAs were changed every 10 days, and participants were advised to wear them for 22 h each day. All patients were instructed to keep the aligners after use and return them monthly to the researchers during appointments. This allowed for the assessment of compliance through blue indicators, which were required for all aligners.
- FA, fixed metallic orthodontic appliance (slot 0.022 x 0.030”, 3M Unitek, Monrovia, USA): the appliances were attached to all of the patients’ teeth. The same sequence of archwires (super elastic nitinol 0.014”, 0.016” and 0.016 x 0.022”) was used for all the patients.

The follow-up was conducted monthly for both groups And the results obtained in the first 12 months were considered.

### Outcome variables (primary and secondary)

#### Severity of crowding and malocclusion

A single examiner assessed the cast models to quantify crowding, based on Little’s irregularity index and using Mitutoyo digital calipers. In addition, the severity of malocclusion was also assessed along with the effects of the proposed orthodontic treatment using peer assessment rating (PAR).^16^

#### OHRQoL

For OHRQoL assessment, study participants answered the Oral Health Impact Profile (OHIP-14) questionnaire,^17,18^which included two questions for each of the seven dimensions, namely: functional limitation; physical pain; psychological discomfort; physical disability; psychological disability; social disability; and overall disability.

The questionnaires were completed before the start of treatment and after 1, 6, and 12 months. OHIP-14 responses were categorized on a five-point scale: Always = 4, Frequently = 3, Sometimes = 2, Rarely = 1, Never or Don’t Know = 0. OHIP point scores were calculated using the additive method, in which the response codes of the 14 items are summed. Consequently, the OHIP-14 scale yielded a total score of 0 to 56, with higher scores indicating worse OHRQoL.^18^ The OHIP-14 is a self-administered questionnaire. The questionnaires were completed by the participants in a private, reserved room at the university, without any assistance. The questionnaires were administered by the same examiner for all participants during the different follow-up periods: before treatment (T0), at 1 month (T1), at 6 months (T2), and at 12 months (T3) after the start of treatment.

#### Sample size

Sample size calculation was based on the study by Drumond-Santana et al.,(19) which identified a mean OHIP-14 standard deviation of 4.8 for healthy patients, . The significance level and statistical power were set at 5% and 80%, respectively, for detection of a minimum difference of five points on the OHIP scale. At least 16 participants were required per group for between-group comparison. To account for potential dropouts, a sample size of 20 individuals per group was established.

#### Interim analyses and stopping guidelines

Not applicable.

#### Randomization

Simple randomization^20^ at a 1:1 ratio was carried out by an external researcher using Excel 2007 (Microsoft Windows, Microsoft, Chicago, IL, USA). The randomization codes were inserted into opaque envelopes, sealed, and consecutively numbered to ensure concealment of group allocations.

#### Blinding

Blinding of participants and operators was not feasible because of the study design. Nevertheless, to enhance study robustness, the results were blindly assessed by assigning code numbers to the participants and groups, thereby minimizing any potential bias introduced by the lack of operator blinding.

## Statistical analysis

To check for intraexaminer reliability in measuring the severity of crowding and malocclusion, 30% of the measurements were repeated and the results were assessed using the intraclass correlation coefficient (ICC) and Bland-Altman agreement, in line with the criteria described by Fleiss.^21^

Statistical analysis was performed using the Statistical Package for Social Sciences (SPSS for Windows, version 22.0, IBM Inc., Armonk, USA). A 5% significance level and a 95% confidence interval were used.

The Shapiro-Wilk test was utilized to assess normality of the data, guiding the choice of the appropriate statistical analysis. Between-group comparisons were conducted using the independent t-test (age, PAR index, Little’s index), the Mann-Whitney test (OHIP-14), and the chi-square test (sex and household income). Friedman’s ANOVA with Bonferroni correction was used for OHIP 14 within-group comparison across the evaluation intervals.

## Results

### Study flowchart

Figure displays the study flowchart, with the evaluation of participants for eligibility, along with randomization, allocation, treatment, and follow-up in the first six months. A total of 54 participants met the inclusion criteria, but only 40 showed an interest in seeking treatment. Orthodontic examinations were conducted in February 2019. After randomization (baseline), participants started treatment in May 2019. During treatment, the appliances were installed, and appropriate instructions were given to the participants. Participants were instructed to return every month for the next 12 months to continue their treatments. One participant was excluded from the OA group for not completing the questionnaire; another patient was excluded from the FA group for relocating to another city, which prevented continuation of treatment.

**Figure:**
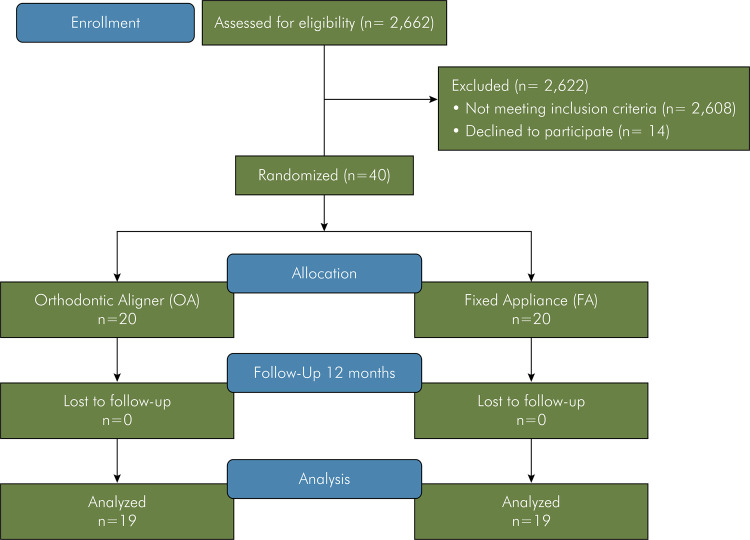
Consolidated Standards of Reporting Trials (CONSORT) flow diagram.

### Baseline data

Participants in both groups were found to be similar in terms of age, sex, cephalometric measurements, degree of crowding (Little’s index), severity of malocclusion (PAR), household income, and OHIP (Table 1).

**Table 1:** Sample compatibility in terms of age, sex, degree of crowding (Little’s Index), severity of malocclusion (PAR index) and family income.

Variables	FA (nv = v19)	OA (nv = v19)	p-value
Age (years) * (Mean/SD)	20.91 (4.35)	23.63 (5.62)	0.100
Sex**			
Male n (%)	12 (75)	12 (66.6)	0.899
Female n (%)	7 (25)	8 (33.4)	
PAR Index (Mean/SD)*	8.26 (3.57)	8.15 (4.89)	0.935
Little’s Index (Mean/SD)*	5.05 (1.91)	4.69 (1.35)	0.502
Family Income**			
Up to 5 minimum salaries n (%)	14 (56.0%)	11 (44.0%)	0.305
> 5 minimum salaries n (%)	5 (38.5%)	8 (61.5%)	

FA: fixed appliance; OA: orthodontic aligner; *Independent t-test; ** Chi-squared test, with Yates’ correction.

### Outcome data

The severity of crowding and malocclusion demonstrated excellent reliability (ICC: Little’s index = 0.98; PAR index = 0.98)(21). Moreover, the Bland-Altman agreement showed low levels of average bias.

Between-group comparison showed that treatment with FA had a significantly higher impact on OHRQoL in the following domains as compared to the OA group: functional limitation (1 month, P=0.025), physical pain (1 month, p = 0.023), psychological discomfort (1 month, p = 0.003), physical disability (1 month, p = 0.046), psychological disability (1 month, p = 0.023), and overall OHIP-14 score (1 month, p = 0.007) (Table 2).

**Table 2:** Oral Health-Related Quality of Life (OHRQoL) at 1, 6, 12 months after starting treatment .

Variables	FA (n =19)	AO (n = 19)	Comparison between groups
	Mean (SD)	Mean (SD)	(p-value)
OHRQoL^*^			
Functional limitation			
1 month	1.74 (1.52)	0.74 (1.09)	0.025*
6 months	0.84 (1.21)	0.68 (1.10)	0.525
12 months	0.95 (1.17)	0.68 (1.20)	0.345
Physical pain			
1 month	4.05 (2.54)	2.25 (1.83)	0.023*
6 months	2.11 (2.02)	2.30 (1.64)	0.678
12 months	2.42 (1.53)	1.60 (1.46)	0.083
Psychological discomfort			
1 month	3.00 (2.13)	1.00 (1.56)	0.003*
6 months	1.37 (1.46)	0.58 (1.17)	0.085
12 months	1.95 (1.95)	1.05 (1.68)	0.091
Physical disability			
1 month	2.79 (2.61)	1.16 (1.83)	0.046*
6 months	1.32 (2.02)	0.58 (0.96)	0.311
12 months	1.11 (1.59)	0.21 (0.71)	0.085
Psychological disability			
1 month	1.95 (1.68)	0.74 (1.09)	0.023*
6 months	0.89 (1.41)	0.53 (0.84)	0.644
12 months	1.11 (1.44)	0.74 (1.32)	0.246
Social disability			
1 month	0.74 (1.24)	0.37 (0.76)	0.506
6 months	0.32 (0.74)	0.26 (0.65)	0.817
12 months	0.53 (1.02)	0.42 (0.76)	0.840
Handicap			
1 month	0.53 (1.64)	0.16 (0.50)	0.776
6 months	0.11 (0.45)	0.11 (0.45)	1.000
12 months	0.16 (0.68)	0.11 (0.45)	1.000
Overall score			
1 month	14.79 (11.01)	6.47 (6.66)	0.007*
6 months	6.84 (6.93)	5.16 (5.32)	0.452
12 months	8.21 (6.86)	4.89 (5.58)	0.091

FA: fixed appliance; OA: orthodontic aligner; *Statistically significant difference (p < 0.05);

Within-group comparison revealed the FA group had a significantly higher mean score in the functional limitation domain at T1 compared to T0 (p = 0.034). The mean score for the physical pain domain was significantly higher at T1 compared to T0 (p = 0.034) and T2 (p = 0.010). In the psychological discomfort domain, the mean score was significantly higher at T1 compared to T2 (p = 0.015); and in the physical disability domain, the mean score was significantly higher at T1 than at T0 (p = 0.008). The mean overall OHIP-14 score was significantly higher at T1 compared to T2 (p = 0.003). No significant changes were observed in the OA group (Table 3).

**Table 3:** Comparison of the means of the OHIP-14 domains and total score during orthodontic treatment.

Groups	Baseline	1 month	6 months	12 months
	Mean (SD)	Mean (SD)	Mean (SD)	Mean (SD)
FA (n=19)				
Functional limitation	0.42 (0.392)^a^	1.74 (1.522)^b^	0.84 (1.214)	0.95 (1.177)
Physical pain	2.05 (1.810)^a^	4.05 (2.549)^b^	2.11 (2.025)^a^	2.42 (1.539)
Psychological discomfort	2.47 (2.038)	3.00 (2.134)^a^	1.37 (1.461)^b^	1.95 (1.957)
Physical disability	0.63 (1.342)^a^	2.79 (2.616)^b^	1.32 (2.029)	1.11 (1.595)
Psychological disability	1.37 (1.707)	1.95 (1.682)	0.89 (1.410)	1.11 (1.449)
Social disability	0.63 (1.300)	0.74 (1.240)	0.32 (0.749)	0.53 (1.020)
Handicap	0.58 (1.071)	0.53 (1.645)	0.11 (0.459)	0.16 (0.688)
Overall score	8.16 (6.938)	14.79 (11.013)^a^	6.84 (6.938)^b^	8.21 (6.868)
OA (n=19)				
Functional limitation	0.32 (0.820)	0.74 (1.098)	0.68 (1.108)	0.68 (1.204)
Physical pain	2.26 (1.790)	2.32 (1.857)	2.42 (1.924)	1.68 (1.455)
Psychological discomfort	1.95 (2.223)	1.00 (1.563)	0.58 (1.170)	1.05 (1.682)
Physical disability	1.42 (1.631)	1.16 (1.834)	0.58 (0.961)	0.21 (0.713)
Psychological disability	1.74 (1.939)	0.74 (1.098)	0.53 (0.841)	0.74 (1.327)
Social disability	0.89 (1.329)	0.37 (0.761)	0.26 (0.653)	0.42 (0.769)
Handicap	0.63 (1.422)	0.16 (0.501)	0.11 (0.459)	0.11 (0.459)
Overall score	9.21 (8.753)	6.47 (6.661)	5.16 (5.326)	4.89 (5.587)

FA: fixed appliance; OA: orthodontic aligners; The groups with the different letters were statistically different (p < 0.05).

3.Sun L, Wong HM, McGrath CP. The factors that influence oral health-related quality of life in young adults. Health Qual Life Outcomes. 2018 Sep;16(1):187. https://doi.org/10.1186/s12955-018-1015-7

## DIscussion

Investigating OHRQoL in orthodontic patients is of fundamental importance for understanding the impact of malocclusion on their daily lives, particularly in terms of functional limitations and psychosocial well-being.^22^ A study that evaluated OHRQoL in adolescents aged 15 to 16 years reported that orthodontic treatment significantly reduced the impact of oral health problems. However, they also stated that orthodontic treatment may have a negative impact on OHRQoL during the course of treatment.^23^

The present study was carried out to gain insight into the impact of OA and FA treatment on OHRQoL during the first 12 months of treatment. Based on patient-centered outcome evaluations, diseases and treatment methods can affect individuals’ social, emotional, and physical aspects. Likewise, subjective evaluations can help define the appropriate treatment goals for each case.^24^

Between-group comparison after 1 month of treatment showed that individuals undergoing orthodontic treatment with FAs demonstrated a significantly higher impact on OHRQoL compared to those treated with OAs. This difference was observed in the context of the following domains: functional limitation, physical pain, psychological discomfort, physical disability, psychological disability, and overall OHIP-14 score. These findings corroborate those of a previous prospective study from China, which investigated the impact of FAs on the OHRQoL of adolescents undergoing orthodontic treatment. The authors reported that the most notable changes occurred in the first week and first month of treatment.^25^ In contrast, an American study evaluating the effects of orthodontic treatment during the first week reported a reduction in OHRQoL in both groups. However, the FA group showed a more pronounced reduction in OHRQoL and a more intense increase in pain compared to the OA group. In the same study, patients in the FA group reported worsening of functional, psychosocial, and pain-related aspects in their daily lives within the first week of treatment.^11^ A Chinese study highlighted the fact that patients reported physical pain, psychological discomfort, and physical disability as recurrent symptoms at the start of treatment. They reported, however, that complaints and the negative impact on OHRQoL decreased as treatment progressed, as observed in the present study.^26^

Within-group comparison revealed that participants from the OA group did not experience significant differences in OHRQoL over the course of 12 months of evaluation. Conversely, patients in the FA group exhibited a significant negative impact in the following domains after the first month of treatment: functional limitation, physical pain, psychological discomfort, physical disability, and overall score. A British prospective study used OHIP-14 to assess patients exclusively treated with FAs throughout the treatment period. The authors reported a negative impact of fixed orthodontic treatment during the first three months, followed by gradual improvement after six months and excellent outcomes by the end of the treatment period. Improvement in OHRQoL was observed in comparison to the pretreatment score. The authors also highlighted several temporary changes in OHRQoL, with significant differences in the following domains: functional limitation, physical pain, psychological discomfort, psychological disability, and social disability.^27^ Similar results were also found by the Chinese prospective study, which evaluated patients treated only with FAs, with a higher negative impact on OHRQoL in the first six months of orthodontic treatment. Similarly, another Chinese study monitored longitudinal changes in OHRQoL during and after treatment with FAs. The authors reported a negative impact on the daily lives of patients, particularly one week after the start of treatment, with significant changes in the domains of physical pain, psychological discomfort, and physical disability. Nevertheless, significant improvement in OHRQoL was observed after the completion of treatment in comparison to the start or progression of treatment.^26^

Although OAs cause less impact on OHRQoL during the initial phase of treatment, evidence suggests that aligners face varying levels of difficulty depending on the type of movement, particularly rotational and vertical movements, which are the most challenging. In more complex cases, aligners may require a longer treatment time to achieve the desired outcomes. The effectiveness of aligners heavily depends on the patient’s commitment to wearing them for the recommended amount of time each day. Frequent removal or improper use can compromise treatment outcomes. Pain perception related to tooth movement appears to have less of an impact at the start of treatment with aligners. Nonetheless, the use of aligners can lead to significant changes in speech and articulation, affecting patient’s communication skills. Aligners are generally more expensive than conventional fixed appliances, which can pose a limitation for some patients.^29^

One of the goals of orthodontic treatment is to improve people’s quality of life through less invasive and more comfortable methods. The literature shows^13,30^ that aligners can provide a better quality of life by causing less masticatory and dietary discomfort compared to fixed appliances. Of note, sociocultural perceptions may vary widely in different parts of the world. Another contentious issue concerns the difference in individual pain perception, as physical pain is considered one of the most influential factors on the quality of life of patients undergoing orthodontic treatment. It is common for patients to experience some discomfort during the adaptation process of orthodontic treatment,^14^ as the appliances can cause pain or even interfere with tongue movements, limiting space for tongue accommodation, consequently affecting patient’s speech.^31^ Our study corroborates this finding, as a decline in quality of life was observed during the first month of treatment with fixed appliances.

The fact that the present study followed the Consort guidelines is also noteworthy. Such guidelines underscore the importance of sample size calculation, inclusion of a control group, and randomization to minimize selection bias, a key component of high-impact RCTs.^15^ A limitation of this study was the lack of follow-up after treatment completion. Given the observed improvement in OHRQoL in the FA group at six months, which persisted after 12 months, the outcomes were unlikely to change by the end of the treatment. Another limitation of our study is the wide age range used. Investigating age subgroups separately might provide a better understanding of changes in OHRQoL during orthodontic treatment with OA and/or FA.

Moreover, at the beginning of the study, the two groups were found to be similar in terms of age, sex, household income, mandibular anterior crowding (Little’s index), and severity of malocclusion (PAR index). These criteria were important for evaluating the impact of orthodontic treatment on OHRQoL. The negative impacts on OHRQoL observed one month after the beginning of treatment in the FA group compared to the OA group are important for helping orthodontists understand the implications of this type of treatment. These findings underscore the importance of considering not just the clinical outcomes but also the impact on patients’ daily lives. Orthodontists can use this information to better counsel patients, preparing them for potential challenges they may face with fixed appliances versus aligners. For instance, while aligners may offer advantages in terms of comfort and aesthetics, there may be some challenges in achieving certain types of tooth movements effectively. On the other hand, fixed appliances may lead to initial discomfort and impact on daily activities such as eating and speaking, which can affect OHRQoL in the short term.

## Conclusion

Patients treated with OAs had less impact on OHRQoL compared to those treated with FAs in the first month of treatment. There was no difference between the groups at the 6-month follow-up. These results highlight the need for orthodontists to carefully evaluate these trade-offs when selecting the appropriate treatment approach for each patient. By discussing these potential impacts at the outset, orthodontists can enhance patient understanding and satisfaction throughout the treatment period, ultimately optimizing both clinical outcomes and quality of life.
